# Devising negative pressure within intercuff space reduces microaspiration

**DOI:** 10.1186/s12871-018-0643-0

**Published:** 2018-12-03

**Authors:** H. M. Sohn, J. S. Baik, J. Y. Hwang, S. Y. Kim, S. H. Han, J. H. Kim

**Affiliations:** 1 Department of Anesthesiology and Pain Medicine, Seoul National University Bundang Hospital, Gyeonggi-do, Republic of Korea; 2 Department of Anesthesiology and Pain Medicine, Pusan National University Hospital, Busan, Republic of Korea; 3 Department of Anesthesiology and Pain Medicine, SMG-SNU Boramae Medical Center, Seoul, Republic of Korea; 4 Department of Anesthesiology and Pain Medicine, Seoul National University Medical College, Seoul, Republic of Korea

**Keywords:** Artificial trachea, Aspiration, Double cuffs, Endotracheal tube, Negative pressure

## Abstract

**Background:**

Microaspiration past the tracheal tube cuffs causes ventilator-associated pneumonia. The objective of the current study was to evaluate whether creating negative pressure between the tracheal double cuffs could block the fluid passage past the tracheal tube cuffs.

**Methods:**

A new negative pressure system was devised between the double cuffs through a suction hole in the intercuff space. Blue-dyed water was instilled above the cuff at negative suction pressures of − 54, − 68, − 82, − 95, − 109, − 122, and − 136 cmH_2_O, and the volume leaked was measured in an underlying water trap after 10 min. Leakage tests were also performed during positive pressure ventilation, and using higher-viscosity materials. The actual negative pressures delivered at the hole of double cuffs were obtained by placing microcatheter tip between the intercuff space and the artificial trachea.

**Results:**

No leakage occurred past the double cuff at − 136 cmH_2_O suction pressure at all tracheal tube cuff pressures. The volume leaked decreased significantly as suction pressure increased. When connected to a mechanical ventilator, no leakage was found at − 54 cmH_2_suction pressure. Volume of the higher-viscosity materials (dynamic viscosity of 63–108 cP <cP> and 370–430 cP) leaked was small compared to that of normal saline (0.9–1.1 cP). The pressures measured in the intercuff space corresponded to 3.8–5.9% of those applied.

**Conclusions:**

A new prototype double cuff with negative pressure in the intercuff space completely prevented water leakage. The negative pressure transmitted to the tracheal inner wall was a small percentage of that applied.

## Background

Aspiration pneumonia in the intensive care unit is a ventilator-related complication that increases antibiotics use, hospital stay, and mortality rates [1, 2]. The incidence of ventilator-associated pneumonia (VAP) accounts for half of hospital-acquired pneumonia and 9–27% of all mechanically ventilated patients [3, 4], whereas mortality rate of VAP ranges from 25 to 50% [5, 6]. The source of aspiration is the accumulation of secretions in the pharynx and upper trachea above tracheal tube cuffs. Any mobilization of pooled secretions can cause a leak along the longitudinal folds of the cuff, which is the most frequent pathogenesis of aspiration [7–9].

Numerous reports have described the attempts to prevent leakage of oropharyngeal secretions past tracheal tube cuffs. Those methods included the use of various cuff shapes and tracheal tube materials [10, 11], applying positive end expiratory pressure to mechanical ventilation [12] or various suction techniques [13, 14], and lubricating the cuff with gel [15].

Since microaspiration of the accumulated secretions around the endotracheal tube cuff is the primary mechanism of VAP, subglottic secretion suctioning has been recommended in several guidelines to avoid the occurrence of VAP [16, 17]. Subglottic secretion suction is known to decrease VAP incidence, reduce duration of mechanical ventilation and delay VAP onset [17, 18].

**Fig. 1:**
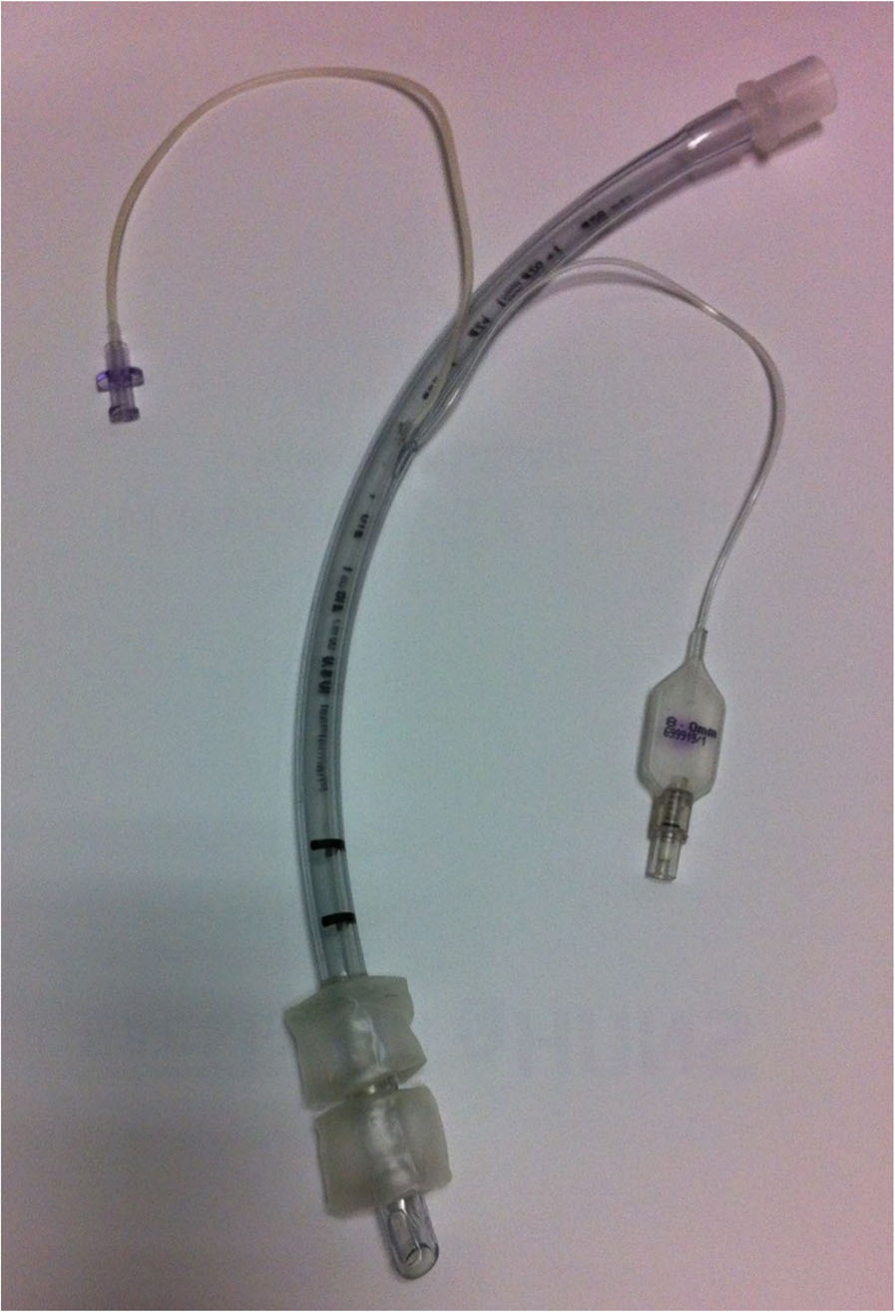
A prototype Double cuff. In the intercuff space, a 1-mm hole is connected to an external port, through which negative suction pressure is applied

Recently, we designed a prototype tracheal tube with double cuffs (Fig. 1), which has the advantage of lowering pressure of two cuffs due to the two volume expansion portions (two cuffs). And between the two cuffs, there is a small hole to either supply a sealing media which can improve trachea sealing, or to provide suction which is subglottic secretion suctioning. We previously reported that an interrupting gel layer between double cuffs prevented fluid leakage in a bench-top model, which method was to use intercuff hole primarily for the purpose of supplying a sealing medium [19]. However, a major limitation of the study was the gel components, which could affect the tracheal wall following prolonged exposure in vivo. Those concerns led us to design a new method of preventing aspiration without using a gel. In the study, the intercuff hole was designed for suctioning, the other main usage of hole between double cuffs. A new negative pressure system was devised between the proximal and distal cuffs—in the intercuff space—to suck the fluid back and forth. The purpose of this study was to investigate whether negative suction pressure between double cuffs would prevent fluid leakage. Specifically, we first investigated whether our intercuff hole could be effectively used for subglottic secretion suction, and then investigated the safety of negative suction by measuring the applied suction pressures.

## Methods

### Tube modifications

We produced a Double cuff tube by attaching two cuffs (proximal and distal) to a standard tracheal tube (Euromedical, Selangor, Malaysia) with an internal diameter of 8.0 mm (Fig. 1). The two cuffs were connected via a small imbedded tunnel in the tracheal tube which enables the cuffs to be inflated consecutively. The new tracheal tube had a small hole (1 mm in diameter) between the cuffs, which was connected to an external port through which negative suction could be applied. Two cylindrical cuffs (28 mm in diameter) were placed 5 mm apart, making the total length of the attached cuffs 45 mm [19].

### Fluid leakage test

**Fig. 2:**
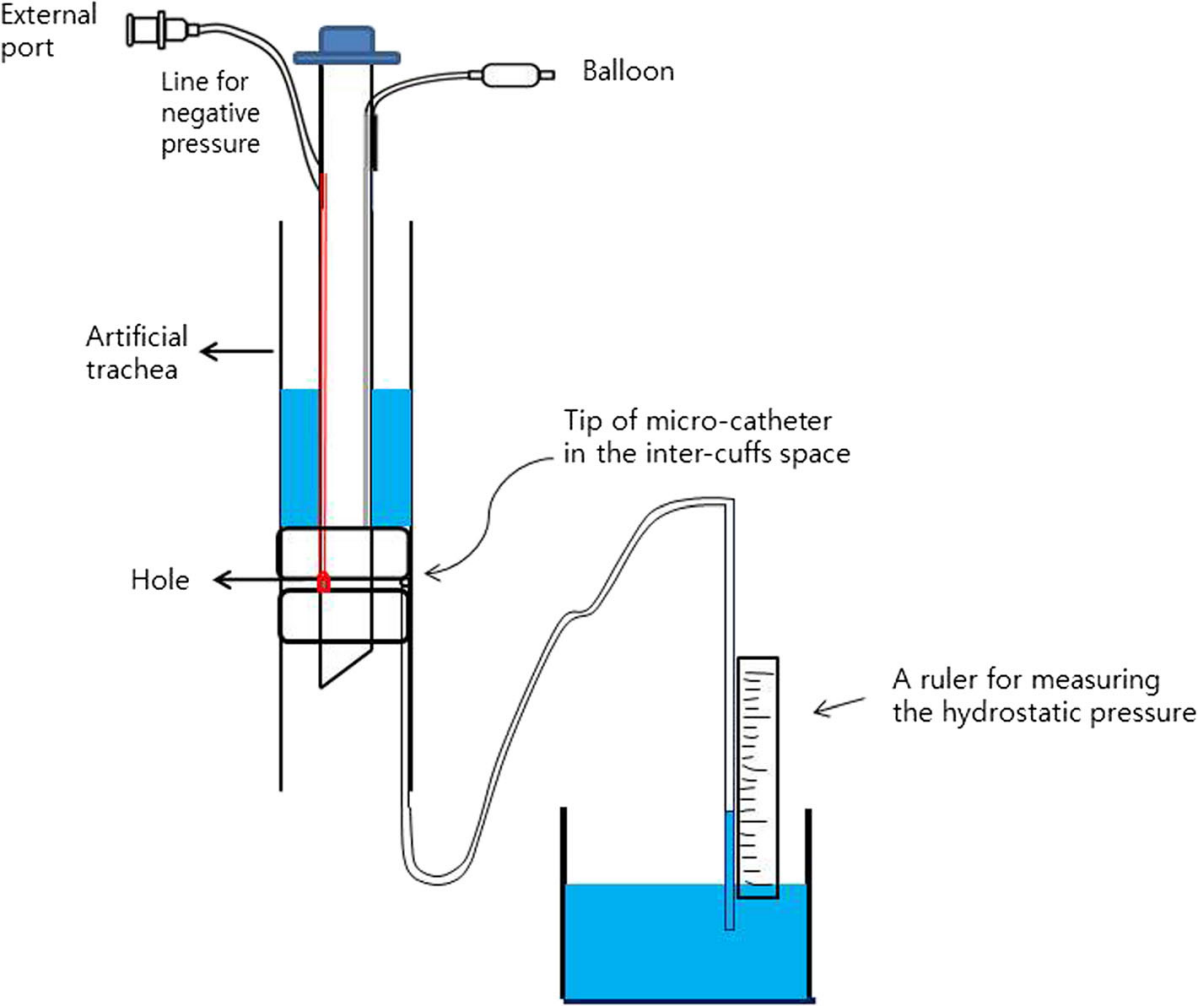
Schematic diagram of leakage testing of the Double cuff

An artificial trachea with a 20-mm internal diameter, corresponding to the size of a human trachea, was intubated with the new tracheal tube or a standard tracheal tube (Euromedical, Selangor, Malaysia) with an internal diameter of 8.0 mm. The lower margin of the standard tracheal tube cuff or the distal cuff of the new tracheal tube was placed 3 cm above the lower edge of the artificial trachea. Methylene-blue-coloured normal saline (dynamic viscosity at 20 °C = 0.9–1.1 cP < cP>) was instilled above the standard tracheal tube cuff or proximal cuff of new tracheal tube to simulate pharyngeal secretions. Any fluid that leaked was collected in an underlying trap, and the volume was measured after 10 min (Fig. 2). Suctioning was delivered using a standard wall suction unit via the external port of the new tracheal tube. Leakage tests were performed at negative suction pressures of − 54, − 68, − 82, − 95, − 109, − 122, and − 136 cmH_2_O, and were repeated five times at each pressure. During the leakage test, the height of instilled saline column above the cuffs was maintained as 3 cm by replenishing the saline; a considerable amount of saline was sucked via the external port of the new tracheal tube (65–110 ml). The both tracheal tube cuffs were inflated together to 15, 20, 25, and 30 cmH_2_O using a hand-held aneroid manometer (VBM, Sulz, Germany) via the pilot balloon.

For mechanical ventilation, the new tracheal tube was connected to a breathing circuit and test lung and then ventilated using the pressure-controlled ventilation mode at an inspiration pressure of 14 cmH_2_O, an FIO_2_ of 0.2, and a frequency of 12/min. Identical experiments were repeated using edible olive oil (dynamic viscosity at 20 °C = 63–108 cP) and a polyoxyethylene sorbitol ester (TWEEN®20, St. Louis, MO, USA) (dynamic viscosity at 20 °C = 370–430 cP) to compare with normal saline at negative pressures of − 41, − 54, − 68, and − 82 cmH_2_O.

Additionally, the actual negative pressure formed between the intercuff space and the tracheal inner wall was assessed. One end of a microcatheter filled with water was placed around the hole of new tracheal tube, and the other end was submerged in a water column. The hydrostatic pressure was measured after application of negative suction pressures of − 50, − 75, − 100, − 150, − 200, and − 300 cmH_2_O through the external port.

### Statistical analysis

All data were assessed for normality using the Kolmogorov–Smirnov test to determine whether parametric or non-parametric statistical tests should be used, and are expressed as means ± standard deviation (SD) or amounts. Mann-Whitney U-test was used to compare the leak volume. A *P*-value < 0.05 was considered significant. Pearson’s correlation coefficient was adopted as a measure of the strength of the relationship between the applied negative pressure and the volume of fluid leaked at each cuff pressure. All statistical analyses were performed using the PASW software (PASW statistics 18.0, SPSS Inc., Munich, Germany).

## Results

**Fig. 3:**
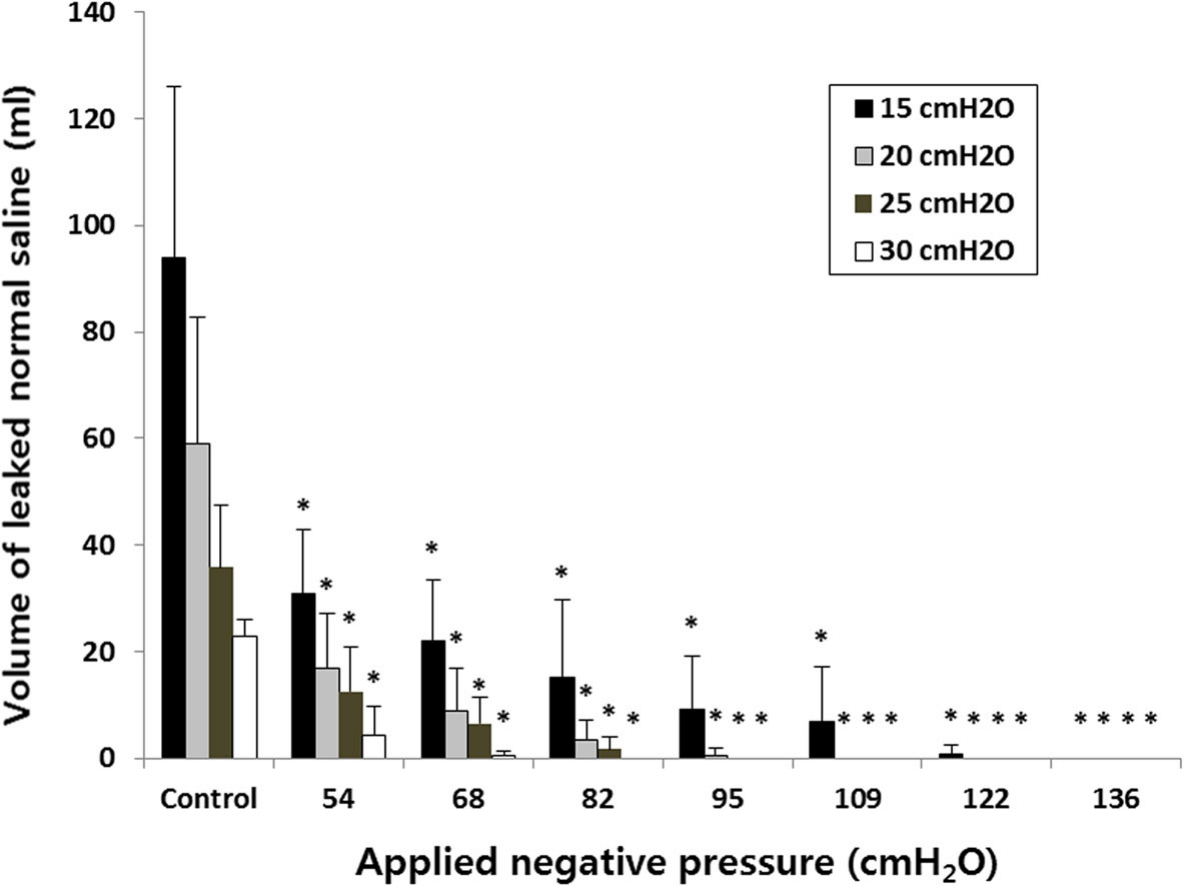
Volume of fluid leaked at different cuff pressures and negative suction pressures for 10 min. The data show a strong inverse relationship between negative pressure and leakage volume. *P < 0.05 vs control. At all negative pressures, the leaked volume from the new tracheal tube were significantly less compared to that of the control

The volumes of saline that leaked past the cuffs during 10 min are shown in Fig. 3. At − 136 cmH_2_O negative suction pressure, no leak was found regardless of the cuff pressure. The negative suction pressures that completely blocked leakage were − 109 cmH_2_O at 20 cmH_2_O cuff pressure, − 95 cmH_2_O at 25 cmH_2_O cuff pressure, and − 82 cmH_2_O at 30 cmH_2_O cuff pressure. The volume leaked decreased significantly with increasing suction pressure at all cuff pressures. At cuff pressures of 15, 20, 25 and 30 cmH_2_O, the Pearson correlation coefficients were − 0.740 (*P* ≤ 0.001), − 0.681 (*P* ≤ 0.001), − 0.668 (*P* ≤ 0.001) and − 0.412 (*P* = 0.014), respectively. At all negative pressures, the amount leaked from the new tracheal tube were significantly less compared to the amount leaked from the standard tracheal tube (*P* < 0.04).

When the mechanical ventilator was applied, no leakage past the double cuffs was observed during a 10-min period, even at − 54 cmH_2_O negative pressure with any cuff pressure (15–30 cmH_2_O, data not shown).

The volume of edible olive oil and polyoxyethylene sorbitol ester that leaked past the cuffs was significantly decreased at all suction pressures at the lowest intracuff pressure (15 cmH_2_O) (Table 1). No occlusion occurred of the intercuff hole or inside the external port during the test.

**Table 1:** Leakage volume of olive oil and polyoxyethylene sorbitol ester (TWEEN® 20) for 10 min at each suction pressure at a cuff pressure of 15 cmH_2_O (*n* = 5)

	41 cmH_2_O	54 cmH_2_O	68 cmH_2_O	82 cmH_2_O
Olive oil	0.39 (0.72)	0.16 (0.36)	0	0
TWEEN®	0	0	0	0

Values are presented as means (sd) (ml)

**Fig. 4:**
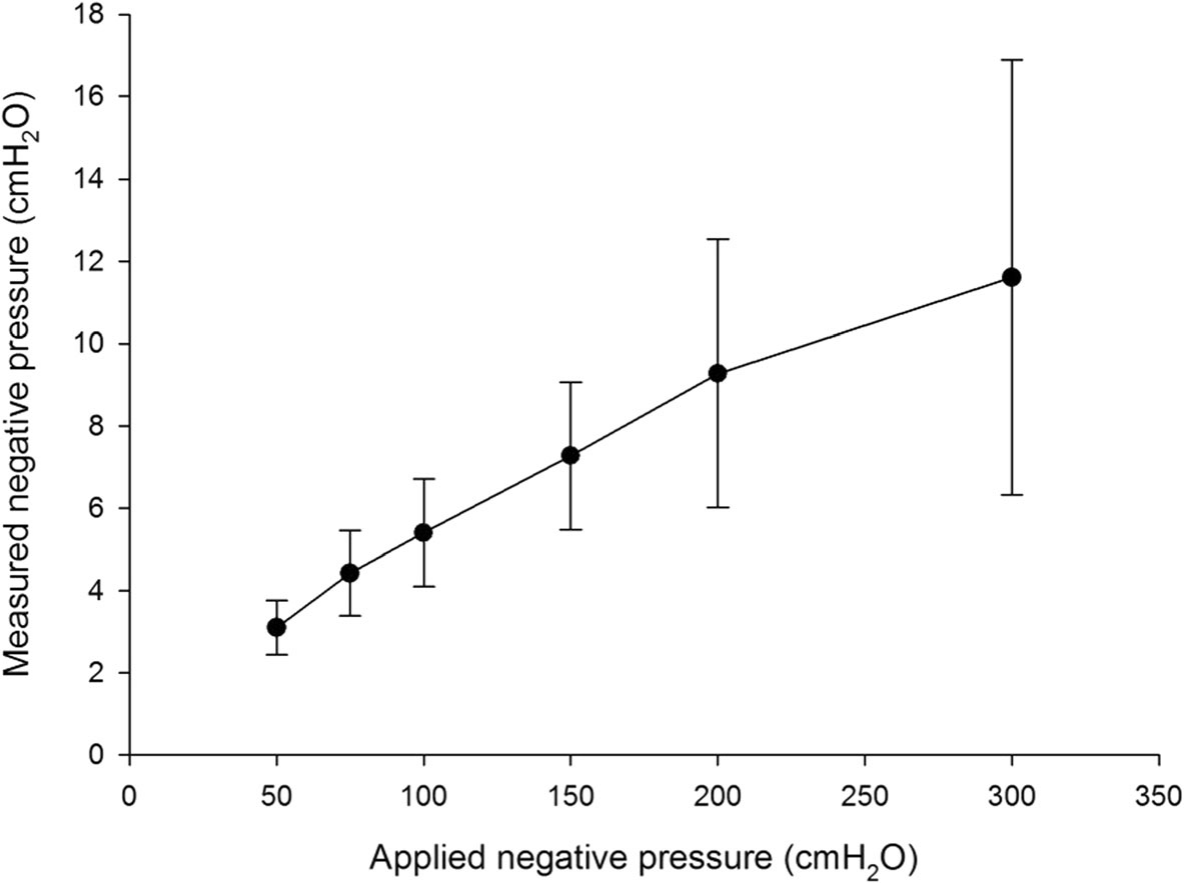
Negative pressures measured between Double cuffs in an artificial trachea according to negative suction pressure. All cuff pressures were 20 cmH_2_O

The measured pressures in the intercuff space corresponded to 3.8–5.9% of the applied negative suction pressures (Fig. 4). When the applied pressure was 300 cmH_2_O, the transmitted measured pressure was 11.5 cmH_2_O.

## Discussion

The present study demonstrated that application of an appropriate negative suction pressure to the intercuff space of the new tracheal tube completely blocked saline leakage past the cuffs. Additionally, the negative pressure measured in the intercuff space corresponded to a small percentage of the applied suction pressure.

Several strategies have been introduced to decrease aspiration pneumonia; however, none has been satisfactory [12, 14, 15]. In a previous study, we demonstrated that a prototype tracheal tube with double cuffs with water-soluble gel in the intercuff space was superior to other endotracheal tubes regarding prevention of leakage in vitro [19]. However, long-term use of gel might cause unexpected effects on the airway, such as irritation and microbial colonisation of the gel layer. Therefore, we aimed to identify a method of blocking microaspiration past the cuffs without using a gel. It is almost impossible to totally prevent leakage around the cuff wall; therefore, outward drainage of fluid that has leaked past the proximal but not the distal cuff may be an effective strategy. Our present results suggest that continuous negative suction between double cuffs may effectively prevent aspiration of subglottic secretions; this intervention would interrupt the key step in the pathogenesis of VAP.

A method that intermittently or continuously suctions secretions in the subglottic area decreases the aspiration risk and prevents VAP [4, 17, 18]. An example of this type of tracheal tube design is the tracheal tube containing an integral suction lumen and available evacuation port—the Hi-Lo Evac (Mallinckrodt, Inc., St. Louis, MO, USA). This type of tracheal tube, which facilitates continuous aspiration of subglottic secretions, has an elliptical dorsal opening above the tracheal tube cuff. The primary purpose of continuous aspiration of subglottic secretions is to aspirate the secretions before they reach the cuff [20]. However, the longitudinal folds of the cuff allow some leakage to pass through the cuff [12]. Accordingly, continuous aspiration of subglottic secretions alone, or proximal aspiration of subglottic secretions is not the solution for complete suction. Unlike conventional drainage systems, in our tube’s drainage system, the drainage of secretions is located below the tracheal tube cuff. The distally-located suction can eliminate the secretions of already microaspirated subglottic secretions through first cuff, which was inevitably created due to incomplete sealing of the tracheal tube cuff. This is the strength of new tracheal tube with double cuffs compared to the tracheal tube with continuous aspiration of subglottic secretions; secretions that flowed downward to the distal cuff flow only after passing both the proximal cuff and the suction. In other words, the new tracheal tube has three barriers (proximal cuff, suction, and distal cuff) for secretion, and can suck down the secretion passed the cuff (the secretion usually to be missed in the proximal suction system).

The negative pressure measured between double cuffs was a small percentage (3.8–5.9%) of that applied. This low intercuff pressure makes our prototype tracheal tube more feasible for use in a clinical setting. The mechanisms underlying the pressure difference need to be identified. One possibility is the resistance caused by the microcatheter between the intercuff hole and the tracheal inner wall. A gap exists between the suction port—on which the regulated vacuum pressure is imposed—and the tracheal inner wall that is in contact with the intercuff space. A pressure drop occurs when frictional forces act on a fluid as it flows through a tube; therefore, the longer the micro catheter is, the larger will be the pressure gradient. Another possibility is that the external negative pressure induces the two cuffs moving closer together, creating small channels in the intercuff space that might dramatically reduce the pressure prior to reaching the trachea. At an applied pressure of 300 cmH_2_O, the measured pressure was 11.5 cmH_2_O, which we consider a permissible safe pressure for in vivo trials.

In the dynamic setup, no leakage was detected at − 54 cmH_2_O negative pressure at intracuff pressures of 15–30 cmH_2_O. Application of positive pressure ventilation to the bench-top model reproduces the impact of mechanical movement on the tube within the trachea. Compared with the static trial, the pneumatic effect generated by the positive pressure distal to the cuff intensified the sealing effect of cuff ballooning. In addition, the positive pressure can push the secretion in the longitudinal fold of the distal cuff (if any) up to the intercuff space, which might improve the removal of the secretion via the negative suction system. This protective pneumatic pressure may permit use of a lower negative pressure to create a complete block. Although positive end expiratory pressure (PEEP) was not adopted in the dynamic study, the PEEP is known to reduce the leak past the cuff of tracheal tube [21]. Therefore, the mechanical ventilation accompanied by PEEP may augment the protective effect of the new tracheal tube to reduce the leakage past the cuffs.

Secretion viscosity affects the development of aspiration pneumonia by influencing bacterial colonization [22, 23]. The subglottic secretions vary in viscosity depending on the circumstances, but whole saliva is usually in the viscosity range of 1.5–3.0 cP [24]. The material used in these tests was normal saline mixed with a small amount of methylene blue, which has a lower viscosity than saliva. It is possible that the volume of leakage would be reduced with more viscous fluids. High-viscosity materials such as olive oil and polyoxyethylene sorbitol ester showed little leakage even at low cuff pressures and did not occlude the suction port or microcatheter. Thick materials can obstruct the slender catheter or small hole. However, any solid, bulky foreign substance is filtered out while passing the primary cuff. Therefore, the suction system operating between the two cuffs only has to remove materials in the form of liquids or small particles. This design may increase the clinical feasibility of the suction system.

This study has several limitations. First, our bench-top system is a crude model of an intubated patient, and we were unable to determine the effects of patient position, patient movement, and differences in cuff inflation along a non-straight trachea. Therefore, we cannot extrapolate the in vitro results to human subjects. Future studies should include in vivo leak and safety testing of a new tracheal tube with double cuffs. Second, the viscosities of olive oil and polyoxyethylene sorbitol ester are considerably higher than human whole saliva; moreover, they are more homogeneous liquids. Thus, more leakage may occur in vivo, and our findings might have differed had subglottic secretions with heterogeneous viscoelastic properties been used. Third, our study has been observed for only 10 min, so it may be said that we have not used enough time to measure fluid leakage more than one hour, for example. However, in practice, in the experiment, water was supplied for a short time of 10 min, but it was a significant amount and there was no aspiration through the lower cuff during this time. In other words, it was judged that the direction or the result of the experiment would not change even if the same experiment lasted more than 10 min. Fourth, like the Hi-Lo Evac (continuous aspiration of subglottic secretions), the new tracheal tube also has the potential for tracheal injury at the level of the suction opening in vivo [25]. Computed tomography has demonstrated invagination of the tracheal mucosa into the suction port of the Hi-Lo Evac, and worrisome mucosal contact [26]. Theoretically, the central drainage hole in the intercuff space creates a gap between the cuffs and the trachea, overcoming the existing problem of direct contact with the mucosa in the present study. However, we used indirect pressure measurements, and more sophisticated methods and in vivo animal study of mucosal injury must be performed to determine the exact pressures exerted on the trachea and the safety of this device. Finally, the length of double cuffs is 4.5 cm. Considering that the adult’s tracheal length is usually 10–11 cm and the tip of the endotracheal tube is recommended to be located at mid-trachea (about 3–7 cm above the carina), the cuff length is too long and impractical for clinical use. Therefore, technical improvements to reduce the cuff length are needed.

## Conclusions

Our prototype tracheal tube with negative pressure between the cuffs completely blocked fluid leakage within a permissible range of suction pressures. Although the pressure measured in the artificial trachea was considerably lower than that applied, for clinical applications, the potential risk of tracheal injury requires further investigation.

## Abbreviations

PEEP: Positive end expiratory pressure
VAP: Ventilator-associated pneumonia
